# The safety and feasibility of comprehensive nutrition care in pregnant women with gestational diabetes mellitus: A protocol for systematic review and meta-analysis

**DOI:** 10.1097/MD.0000000000025505

**Published:** 2021-05-07

**Authors:** Mei Long, Xiangchun Zhang

**Affiliations:** Department of Obstetrics and Gynecology, Hubei Huanggang Central Hospital, Hubei Province, China.

**Keywords:** comprehensive nutritional care, gestational diabetes mellitus, medical nutritional therapy, meta-analysis, protocol, systematic review

## Abstract

**Background:**

The argument on the efficacy of medical nutritional therapy and comprehensive nutritional care remains to be resolved. Therefore, we conducted this protocol of systematic review and meta-analysis to evaluate the efficacy between medical nutritional therapy and comprehensive nutritional care for patients with gestational diabetes mellitus (GDM).

**Methods:**

We followed the Preferred Reporting Items for Systematic Reviews and Meta-Analyses Protocols reporting guidelines and the recommendations of the Cochrane Collaboration to conduct this study. Reviewers will search the PubMed, Cochrane Library, Web of Science, and EMBASE online databases using the key phrases “gestational diabetes mellitus,” “comprehensive nutrition care,” and “medical nutritional therapy” for all cohort studies published up to May 20, 2021. There is no restriction in the dates of publication or language in the search for the current review. The studies on cohort study focusing on comparing medical nutritional therapy and comprehensive nutrition care for GDM patients will be included in our meta-analysis. The outcomes include blood glucose levels, complications, weight change, and incidence of cesarean section. Where disagreement in the collection of data occurrs, this will be resolved through discussion.

**Results:**

We hypothesized that these 2 methods would provide similar therapeutic benefits.

**OSF registration number:**

10.17605/OSF.IO/SC8HJ.

## 1 Introduction

Gestational diabetes mellitus (GDM) is defined as glucose intolerance that occurs during pregnancy. As with type 2 diabetes mellitus, the incidence of GDM is increasing. GDM currently affects about 5% to 10% of pregnancies in the United States, with about 250,000 new cases each year.^[1]^ Not only has the prevalence of GDM been steadily increasing over the past 20 years, but national trends of advanced maternal age, obesity, and reduced physical activity are also increasing, which will lead to further increases in the prevalence of GDM in the coming years.^[2,3]^

GDM is associated with an increased risk of adverse fetal, infant, and maternal pregnancy outcomes, including primary caesarean delivery, preeclampsia, fetal hyperplasia, and neonatal hypoglycemia.^[4–6]^ Although the hyperglycemia of GDM usually disappears after delivery, women with GDM have an increased risk of further episodes of GDM and are 7 times more likely to develop type 2 diabetes mellitus.^[7]^ In addition, there is growing evidence that hyperglycemia during pregnancy can program the long-term metabolic health of offspring, thereby increasing their risk of developing type 2 diabetes mellitus.^[8–10]^ At present, there is no effective treatment for the treatment of GDM. Therefore, a new intervention is urgently needed.

Medical nutritional therapy is the basic treatment for GDM and its comorbidities. The goal of medical nutritional therapy in patients with GDM is to encourage the adoption of a healthy diet to achieve and maintain normoglycemia and to promote adequate gestational weight gain and appropriate fetal growth.^[11]^ Despite the importance of medical nutritional therapy for GDM management, it also has obvious shortcomings, including the lack of follow-ups.^[12]^ Comprehensive nutritional care is a new method created in recent years to treat GDM. It consists of a nutritious diet, postprandial exercise, health education, and regular follow-up, and is considered a new intervention to replace medical nutrition therapy.^[13]^ However, the argument on the efficacy of medical nutritional therapy and comprehensive nutritional care remains to be resolved. Therefore, we conducted this protocol of systematic review and meta-analysis to evaluate the efficacy between medical nutritional therapy and comprehensive nutritional care for patients with GDM.

## 2 Materials and methods

### 2.1 Search strategy

We followed the Preferred Reporting Items for Systematic Reviews and Meta-Analyses Protocols reporting guidelines and the recommendations of the Cochrane Collaboration to conduct this study. Reviewers will search the PubMed, Cochrane Library, Web of Science, and EMBASE online databases using the key phrases “gestational diabetes mellitus,” “comprehensive nutrition care,” and “medical nutritional therapy” for all cohort studies published up to May 20, 2021. There is no restriction in the dates of publication or language in the search for the current review, and thus publication and language bias can be minimized. Ethical approval is not necessary because the present meta-analysis will be performed based on previous published studies. The prospective registration has been approved by the Open Science Framework registries (with the number 10.17605/OSF.IO/SC8HJ).

### 2.2 Inclusion and exclusion criteria

The studies on cohort study focusing on comparing medical nutritional therapy and comprehensive nutrition care for GDM patients will be included in our meta-analysis. At least one of the following outcomes should have been measured: blood glucose levels, complications, weight change, and incidence of cesarean section. The exclusion criteria contain biochemical trials, reviews, case reports, no assessment of outcomes mentioned above, and no comparison of medical nutritional therapy and comprehensive nutrition care.

### 2.3 Study selection

Two independent authors will follow the unified search strategy to screen the titles and abstracts of potentially relevant studies. Any inconsistencies between reviewers will be resolved through discussion and consensus. If a consensus can not be reached, a senior author will be consulted for a final decision.

### 2.4 Data extraction

The method of data extraction will follow the approach outlined by the Cochrane Handbook for Systematic Reviews of Interventions. Two independent authors extract the following descriptive raw information from the selected studies: study characteristics such as the first author, publication year, study design, follow-up period; patient demographic details such as patients’ number, average age, and sex ratio. The outcomes include blood glucose levels, complications, weight change, and incidence of cesarean section. Where disagreement in the collection of data occurrs, this will be resolved through discussion. The corresponding author will be contacted and asked to provide the data if the SD is not reported. In the case of no response, the SD is calculated from the available data according to the previously validated formula: (higher range value – lower range value)/4 or interquartile range/1.35. The highest SD is used if the SD cannot be calculated using this approach. If necessary, we will abandon the extraction of incomplete data.

### 2.5 Methodological quality assessment

The Cochrane risk of bias tool^[12]^ is used to evaluate the risk of bias of included RCTs by 2 independent reviewers. The quality of RCTs is assessed by using following 7 items: random sequence generation, allocation concealment, blinding of participants and personnel, blinding of outcome assessment, incomplete outcome data, selective reporting, and other bias. Disagreement is resolved through discussion and consensus between the reviewers. Kappa values will be used to measure the degree of agreement between the 2 reviewers and are rated as follows: fair, 0.40 to 0.59; good, 0.60 to 0.74; and excellent, 0.75 or more. Based on the information provided from included studies, each item is recorded as low risk of bias, high risk of bias, or unclear (lack of information or unknown risk of bias). We also conduct the sensitivity analysis to evaluate whether any single study has the weight to skew on the overall estimate and data. Begg funnel plot is used to assess publication bias. If publication bias exists, the Begg funnel plot is asymmetric.

### 2.6 Data analysis

Review Manager software (v 5.3; Cochrane Collaboration) is used for the meta-analysis. Extracted data are entered into Review Manager by the first independent author and checked by the second independent author. Risk ratio with a 95% confidence interval or standardized mean difference with 95% CI are assessed for dichotomous outcomes or continuous outcomes, respectively. The heterogeneity is assessed by using the *Q* test and *I*^2^ statistic. An *I*^2^ value of <25% is chosen to represent low heterogeneity and an *I*^2^ value of >75% to indicate high heterogeneity. All outcomes are pooled on random-effect model. A *P* value of <.05 is considered to be statistically significant.

## 3 Discussion

To the best of our knowledge, this is the first study to date to compare the efficacy between medical nutritional therapy and comprehensive nutritional care for patients with GDM. We hypothesized that these 2 methods would provide similar therapeutic benefits. We conducted this systematic review and meta-analysis according to the Preferred Reporting Items for Systematic Reviews and Meta-Analyses Protocols guidelines. Two independent authors will use a highly sensitive search strategy to identify the trials in the main databases and supplement it by manually searching for studies related to the topic and the reference list of included studies. There is no restriction in the dates of publication or language in the search for the current review, and thus publication and language bias can be minimized. In accordance with recommendations of GRADE, the quality of the evidence will be carefully evaulated in this review, and thus generating a precise level of confidence of our results.

## Author contributions

**Conceptualization:** Xiangchun Zhang.

**Data curation:** Mei Long, Xiangchun Zhang.

**Formal analysis:** Mei Long.

**Funding acquisition:** Xiangchun Zhang.

**Investigation:** Mei Long, Xiangchun Zhang.

**Methodology:** Mei Long, Xiangchun Zhang.

**Resources:** Xiangchun Zhang.

**Software:** Mei Long, Xiangchun Zhang.

**Supervision:** Mei Long.

**Writing – original draft:** Mei Long, Xiangchun Zhang.

**Writing – review & editing:** Xiangchun Zhang.

## References

[1] Martinez NG Niznik CM Yee LM. Optimizing postpartum care for the patient with gestational diabetes mellitus. Am J Obstet Gynecol 2017;217:314–21. 28455081 10.1016/j.ajog.2017.04.033 PMC5581263

[2] Li YX Long DL Liu J. Gestational diabetes mellitus in women increased the risk of neonatal infection via inflammation and autophagy in the placenta. Medicine (Baltimore) 2020;99:e22152. 33019392 10.1097/MD.0000000000022152 PMC7535644

[3] Parsons J Sparrow K Ismail K. Experiences of gestational diabetes and gestational diabetes care: a focus group and interview study. BMC Pregnancy Childbirth 2018;18:25. 29325518 10.1186/s12884-018-1657-9 PMC5765597

[4] Zhu Y Zhang C. Prevalence of gestational diabetes and risk of progression to type 2 diabetes: a global perspective. Curr Diab Rep 2016;16:07. 10.1007/s11892-015-0699-x PMC6675405 26742932

[5] Ferrara A. Increasing prevalence of gestational diabetes mellitus. Diabetes Care 2007;30:S141–6. 17596462 10.2337/dc07-s206

[6] HAPO Study Cooperative Research Group. Hyperglycemia and adverse pregnancy outcomes. N Engl J Med 2008;2008:1991–2002. 10.1056/NEJMoa0707943 18463375

[7] Kim C Berger DK Chamany S. Recurrence of gestational diabetes mellitus. Diabetes Care 2007;30:1314–9. 17290037 10.2337/dc06-2517

[8] Bellamy L Casas J-P Hingorani AD. Type 2 diabetes mellitus after gestational diabetes: a systematic review and meta-analysis. Lancet 2009;373:1773–9. 19465232 10.1016/S0140-6736(09)60731-5

[9] Dabelea D Knowler WC Pettitt DJ. Effect of diabetes in pregnancy on offspring: follow-up research in the pima Indians. J Matern Fetal Med 2000;9:83–8. 10757442 10.1002/(SICI)1520-6661(200001/02)9:1<83::AID-MFM17>3.0.CO;2-O

[10] Krishnaveni GV Veena SR Hill JC. Intrauterine exposure to maternal diabetes is associated with higher adiposity and insulin resistance and clustering of cardiovascular risk markers in Indian children. Diabetes Care 2010;33:402–4. 19918007 10.2337/dc09-1393 PMC2809291

[11] Tsirou E Grammatikopoulou MG Theodoridis X. Guidelines for medical nutrition therapy in gestational diabetes mellitus: systematic review and critical appraisal. J Acad Nutr Diet 2019;119:1320–39. 31201104 10.1016/j.jand.2019.04.002

[12] American Diabetes Association. Management of diabetes in pregnancy: Standards of medical care in diabetes—2018. Diabetes Care 2018;41:S137–43. 29222384 10.2337/dc18-S013

[13] Yuan K Wang H Chen Y. A 12-hour comprehensive nutrition care benefits blood glucose level and weight gain and improves outcomes in pregnant women with gestational diabetes mellitus. Ann Palliat Med 2020;9:661–70. 32279518 10.21037/apm.2020.03.16

